# *Notes from the Field:* Group A *Streptococcus* Bacteremia in Persons Who Inject Drugs — Northern Vermont, January 2020–October 2023

**DOI:** 10.15585/mmwr.mm7316a5

**Published:** 2024-04-25

**Authors:** Monica J. Raymond, Tonda R. Wolfe, Lindsay M. Smith

**Affiliations:** ^1^University of Vermont Medical Center, Burlington, Vermont; ^2^Larner College of Medicine, University of Vermont, Burlington, Vermont.

**Summary**

**What is already known about this topic?**

Injection of illicit drugs and homelessness are risk factors for invasive group A streptococcal infections. Xylazine has been associated with necrosis, which could facilitate entry of bacteria into the bloodstream.

**What is added by this report?**

During 2022–2023, the University of Vermont Medical Center experienced a substantial increase in the number of community-acquired group A streptococcal bloodstream infections, predominantly in persons who inject drugs. The increase coincided with the introduction of xylazine into the drug supply. Many patients sought care for wounds before being diagnosed with a bloodstream infection.

**What are the implications for public health practice?**

The availability of wound care services in sites accessible to persons who inject drugs might help prevent bloodstream infections.

CDC has recently reported increases in invasive group A *Streptococcus* (GAS) infections.* Injection of illicit drugs and homelessness are two documented risk factors for invasive GAS infections (*1*). In 2022 and 2023, the University of Vermont Medical Center (UVMMC) Infection Prevention and Antimicrobial Stewardship programs detected a substantial increase compared with 2020–2021 in community-acquired GAS bacteremia among adult patients seeking care at UVMMC. The programs conducted an investigation to identify opportunities to enhance the delivery of care.

## Investigation and Outcomes

Cases of invasive GAS infections were identified using reports in the electronic medical record (EMR), using Epic software. A case of GAS bacteremia was defined as *Streptococcus pyogenes* in a blood culture from a UVMMC patient during January 1, 2020–October 31, 2023. A repeat positive culture occurring >30 days after the initial positive culture was considered a recurrent infection and was included in the analysis. Patients meeting the following criteria were excluded: those who transferred to UVMMC with GAS bacteremia, those who had been admitted to the hospital during the previous 7 days, and those whose initial positive culture specimen was obtained ≥48 hours after hospital admission, ≤7 days after surgery, or ≤7 days postpartum. As a quality improvement project aimed at identifying risk factors, developing prevention strategies, and improving patient care for GAS bacteremia, this activity did not require institutional review board review.

Among UVMMC patients, three cases of GAS bacteremia were identified in 2020, four in 2021, 19 in 2022, and 45 during the first 10 months of 2023 (Figure). In comparison, total emergency department patient encounters at UVMMC increased by 19% between 2020–2021 and 2022–2023, and total admissions increased by <2%.

**FIGURE:**
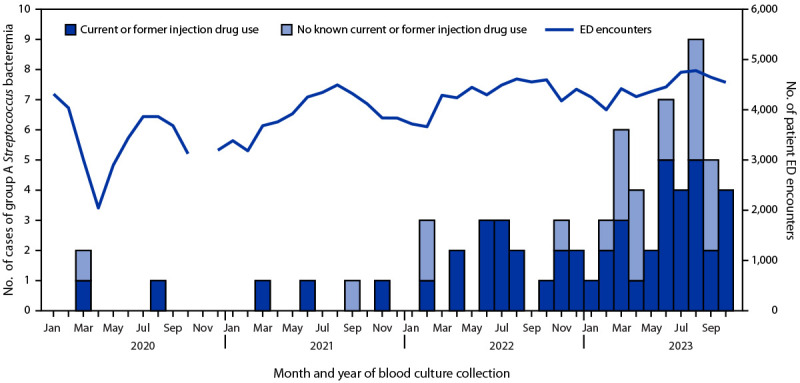
Cases of community-acquired group A *Streptococcus* bacteremia, by month of blood culture collection, patient history of injection drug use, and emergency department encounters — University of Vermont Medical Center, January 2020–October 2023*^,^^†^ **Abbreviation:** ED = emergency department. * Infection and ED encounter data are missing for October 28–November 23, 2020, because of a cyberattack that rendered the University of Vermont Medical Center’s electronic medical records unusable. ^†^ In October 2021, the Vermont Department of Health reported a greater than twofold increase in the percentage of fatal opioid overdoses with xylazine involvement during the first 7 months of 2021, compared with each of the previous 2 years.

Of the 64 cases identified during 2022–2023, a total of 45 (70%) occurred among 38 patients known to be persons who inject drugs^†^ (PWID), based on self-report documented in the EMR. The remainder of the report focuses on these 38 persons with 45 cases of GAS bacteremia.

Twenty-one (55%) of the 38 patients were female; median patient age was 40.5 years (range = 22–63 years). Among 28 (62%) of the 45 cases, the patient reported experiencing homelessness at the time of GAS bacteremia diagnosis, compared with one of the 19 cases among non-PWID. Among 35 (78%) cases, patients reported active injection drug use at the time of bacteremia; among the remaining 10 (22%) cases, patients reported previous injection drug use and current noninjection illicit drug use. Known xylazine exposure before diagnosis was self-reported in 12 (27%) cases and suspected by a clinician based on the presence of wounds consistent with xylazine use (*2*) in an additional seven (16%) cases.

Among 44 of the 45 cases, the patients had concurrent skin and soft tissue infections; in 37 (82%) cases, the patients had multiple wounds at the time of diagnosis with GAS bacteremia. Twenty-one of the 38 patients collectively sought aid 59 times (range = one to six visits per person) at UVMMC emergency or urgent care departments for wound care during the 6 months before their diagnosis of GAS bacteremia.

Hospital admission for intravenous antibiotic therapy was recommended for all cases. Among 17 (38%) cases, the patient underwent wound debridement (12 in an operating room and five at bedside). Among 23 (51%) cases, the patient declined admission or left the hospital against medical advice. The average length of admission was 11 days. Two patients died during hospitalization for GAS bacteremia.

## Preliminary Conclusions and Actions

The precipitous increase in GAS bacteremia at UVMMC followed an increase in involvement of xylazine in fatal opioid overdoses in Vermont, first reported in late 2021.^§^^,^^¶^ Xylazine causes peripheral vasoconstriction and ischemia and has been associated with necrosis at injection sites and noninjection sites (*2*). Xylazine can be present in both injected and noninjected drugs.** Xylazine-related wounds might serve as a portal of entry for bacteria into the bloodstream and could, at least in part, explain the increase in GAS bacteremia described in this report. Given the findings of this report and other studies (*1*), GAS should be considered in PWID with symptoms of bacteremia, particularly in persons with wounds. During the 6 months before diagnosis with GAS bacteremia, patients visited emergency or urgent care departments up to six times seeking aid for wound care. Increased access to wound care services in sites accessible to PWID might result in earlier treatment and prevent progression to bacteremia.

In response to these findings, UVMMC is working to improve linkage to care for both opioid use disorder and wound care and is exploring collaborative efforts with local nongovernmental organizations and public health authorities to deliver wound care services in community settings.

## Footnotes

*
https://www.cdc.gov/groupastrep/current-activity.html

† Includes persons who actively engaged in injection drug use at the time of GAS bacteremia diagnosis and those who did so previously.

§
https://www.healthvermont.gov/sites/default/files/documents/pdf/ADAP-XylazineBrief.pdf

¶
https://www.cdc.gov/drugoverdose/fatal/dashboard/index.html

**
https://www.cdc.gov/drugoverdose/deaths/other-drugs/xylazine/faq.html#what
